# Improved motor imagery classification using adaptive spatial filters based on particle swarm optimization algorithm

**DOI:** 10.3389/fnins.2023.1303648

**Published:** 2023-12-13

**Authors:** Xiong Xiong, Ying Wang, Tianyuan Song, Jinguo Huang, Guixia Kang

**Affiliations:** ^1^School of Information and Communication Engineering, Beijing University of Posts and Telecommunications, Beijing, China; ^2^Department of Neuroscience, University of Sheffield, Sheffield, United Kingdom; ^3^School of Automation, Beijing University of Posts and Telecommunications, Beijing, China

**Keywords:** MI-EEG, brain computer Interface, common spatial pattern, particle swarm optimization, spatial filters

## Abstract

**Background:**

As a typical self-paced brain–computer interface (BCI) system, the motor imagery (MI) BCI has been widely applied in fields such as robot control, stroke rehabilitation, and assistance for patients with stroke or spinal cord injury. Many studies have focused on the traditional spatial filters obtained through the common spatial pattern (CSP) method. However, the CSP method can only obtain fixed spatial filters for specific input signals. In addition, the CSP method only focuses on the variance difference of two types of electroencephalogram (EEG) signals, so the decoding ability of EEG signals is limited.

**Methods:**

To make up for these deficiencies, this study introduces a novel spatial filter-solving paradigm named adaptive spatial pattern (ASP), which aims to minimize the energy intra-class matrix and maximize the inter-class matrix of MI-EEG after spatial filtering. The filter bank adaptive and common spatial pattern (FBACSP), our proposed method for MI-EEG decoding, amalgamates ASP spatial filters with CSP features across multiple frequency bands. Through a dual-stage feature selection strategy, it employs the Particle Swarm Optimization algorithm for spatial filter optimization, surpassing traditional CSP approaches in MI classification. To streamline feature sets and enhance recognition efficiency, it first prunes CSP features in each frequency band using mutual information, followed by merging these with ASP features.

**Results:**

Comparative experiments are conducted on two public datasets (2a and 2b) from BCI competition IV, which show the outstanding average recognition accuracy of FBACSP. The classification accuracy of the proposed method has reached 74.61 and 81.19% on datasets 2a and 2b, respectively. Compared with the baseline algorithm, filter bank common spatial pattern (FBCSP), the proposed algorithm improves by 11.44 and 7.11% on two datasets, respectively (*p* < 0.05).

**Conclusion:**

It is demonstrated that FBACSP has a strong ability to decode MI-EEG. In addition, the analysis based on mutual information, t-SNE, and Shapley values further proves that ASP features have excellent decoding ability for MI-EEG signals and explains the improvement of classification performance by the introduction of ASP features. These findings may provide useful information to optimize EEG-based BCI systems and further improve the performance of non-invasive BCI.

## 1 Introduction

Brain–computer interface (BCI) technology is an emerging field that allows direct connection between the brain and external devices (Zimmermann-Schlatter et al., 2008; Tonin and Millán, 2021). BCIs have many potential applications, including assisting paralyzed patients, operating machines in extreme environments, and controlling neuroprosthetic limbs (Göhring et al., 2013; Baniqued et al., 2021; Moioli et al., 2021). Non-invasive electroencephalogram (EEG) signals break away from the ethical constraints and the requirements of invasive brain surgery and become a more suitable way to construct BCI for normal people (Kauhanen et al., 2006; Long et al., 2010; Ahangi et al., 2013; Gu and Chou, 2021). For non-invasive brain–computer interfaces, sensorimotor rhythms (Cook et al., 2021), event-related potentials (Doan et al., 2021), and steady-state visual evoked potentials (Zhao et al., 2021) are the three main application paradigms. Motor imagery (MI) is a common method used by humans to evoke sensorimotor rhythms in an autonomous way (Hétu et al., 2013; Hardwick et al., 2018). Motor imagery tasks inhibit contralateral sensorimotor areas of the brain. MI-based systems have shown great potential in helping patients with stroke (Mane et al., 2020; Yang et al., 2021), spinal cord injuries (Olsson, 2012; Athanasiou et al., 2018; Pulferer et al., 2022), and amyotrophic lateral sclerosis (Stanton et al., 2007; Kasahara et al., 2012).

For MI-BCI, accurate decoding of user intentions is crucial for the practicability and robustness of BCI systems. Explaining effective features related to motor imagery in EEG signals is the key to accurate decoding (Zimmermann-Schlatter et al., 2008; Long et al., 2010). Some researchers have used event-related desynchronization and event-related synchronization (ERD/ERS) to classify mental states (Quiroga et al., 2007; Yeom and Sim, 2008). However, the low signal-to-noise ratio (SNR) of EEG signals affects the detection of ERD/ERS patterns. In addition, due to the topology of motor neurons, the EEG signals collected from the cerebral cortex are usually mixed by multiple sensorimotor neurons, resulting in poor spatial resolution of the original EEG signals and reducing pattern recognition performance (Chen et al., 2021).

To improve the spatial resolution of ERD suppression and ensure the performance of pattern recognition, commonly used feature extraction methods include spectral analysis (Hou et al., 2022), autoregressive (Jafarifarmand et al., 2017), source reconstruction (Salazar-Varas and Vazquez, 2019), and common spatial pattern (CSP) (Lee et al., 2019). Among them, CSP features have been widely used in MI-BCI. The algorithm transforms the EEG signal by solving an optimal spatial filter to maximize the variance of one MI task and minimize the variance of the other MI task. Therefore, the CSP algorithm is suitable for feature extraction of multi-variable EEG signals (Müller-Gerking et al., 1999).

However, the traditional CSP features have the problem of over-fitting, so some methods improve the effect of the CSP algorithm by weighting or regularizing CSP features. Regularized Common Spatial Pattern (RCSP) improves classification accuracy by combining CSP with ridge regression and regularization (Lu et al., 2010). Furthermore, the filter bank regularized common spatial pattern (FBRCSP) introduces a filter bank based on RCSP and uses feature selection based on mutual information to reduce the dimension to improve the recognition effect of MI. Discriminative FBCSP (DFBCSP) achieves high classification performance by combining FBCSP with discriminative classifiers (Higashi and Tanaka, 2013). Sparsity FBCSP (SFBCSP) incorporates sparsity constraints into FBCSP to enhance feature selection and reduce feature space dimension (Zhang et al., 2015). Furthermore, the local region frequency CSP (LRFCSP) extracts features from specific frequency bands in local brain regions to improve classification accuracy (Park and Chung, 2019). Spectrally weighted CSP (SWCSP) weights the contribution of different frequency bands by considering the correlation of frequency features (Meng et al., 2013). Penalized time-frequency band CSP (PTFBCSP) is similar to SWCSP by penalizing irrelevant features to improve classification accuracy, but PTFBCSP further considers irrelevant features in time and frequency domains (Peterson et al., 2019).

In addition, some studies consider both sequence relationships and frequency bands to enhance CSP. For example, the most representative research includes Separable CSSP (SCSSP), which improves classification accuracy by simultaneously considering sequence relationships and frequency bands in EEG signals (Aghaei et al., 2015). CSP based on the longest continuous repeated sliding window (LCR-SW-CSP) improves CSP features through multiple time windows, thereby enhancing the classification accuracy when processing MI-EEG (Gaur et al., 2021). Temporal-constrained sparse group spatial pattern (TSGSP) uses temporal-constrained sparse constraints to extract spatial features (Zhang et al., 2018). Time-frequency CSP (TFCSP) extracts CSP features in the time-frequency domain to obtain more effective features (Mishuhina and Jiang, 2021).

Furthermore, other studies enhance or extend CSP features to improve classification accuracy. The most commonly used method in this category to enhance CSP features is to extract spatial features while considering frequency bands. The most representative research is the filter bank CSP (FBCSP) (Ang et al., 2008). FBCSP obtains CSP features over different frequency bands by introducing filter banks into EEG signals before using CSP feature extraction. There are some other methods that also consider frequency domain information, including common spatial-spectral pattern (CSSP), which considers both spatial and spectral information in EEG signals (Lemm et al., 2005). CSSP aims to identify a set of spatial filters that can capture spatial and spectral features specific to a given task or class. To achieve better results, these methods extend and expand the features that the CSP method can extract from EEG signals.

However, the above methods usually do not modify the CSP algorithm itself. Neither the shift in the frequency band or the time window nor the addition of some regularization will change the purpose of the CSP algorithm, which is to distinguish the variance of the two classes of EEG signals by finding spatial filters (Marini and Walczak, 2015).

To make up for these deficiencies, this study introduces a new spatial filter-solving paradigm, which is named adaptive spatial pattern (ASP). Unlike CSP, which wants to make the variance difference between the two types of MI-EEG larger, ASP aims to minimize the energy intra-class matrix and maximize the inter-class matrix of MI-EEG after spatial filtering. The inter-class matrix quantifies the spread between different class categories, aiming to maximize class separability, while the intra-class matrix characterizes the dispersion of data points within the same class, emphasizing data compactness. ASP’s primary objective is to minimize the energy intra-class matrix while simultaneously maximizing the inter-class matrix of MI-EEG following spatial filtering. This unique strategy empowers ASP to discern the total energy distribution across various frequency bands in EEG signals. By minimizing the energy intra-class matrix, ASP aims to make the energy distribution more uniform within each class, thus addressing the limitations of CSP’s emphasis on variance differences. At the same time, maximizing the “inter-class matrix” increases the separation between different classes, resulting in improved discrimination capabilities. Therefore, ASP can distinguish the total energy of EEG signals in each frequency band as a supplement to the spatial filter obtained by the CSP algorithm. To solve ASP, we use the local best particle swarm optimization algorithm to compute through continuous iteration. For combining ASP features and CSP features, a reasonable algorithm framework is designed (FBACSP). The proposed FBACSP algorithm can not only select the subject-specific optimal spatial filter to improve the accuracy of MI-BCI classification but also reduce the feature dimension for different subjects and suppress the negative impact of noise.

The contributions of this study are listed as follows:

1. For decoding MI-EEG, a new spatial filter named ASP is defined, which aims to distinguish the overall energy characteristics of different types of MI-EEG, and the solution process adopts a local optimal particle swarm optimization algorithm.
2. To improve the classification efficiency, the redundant features unrelated to MI are dropped. A two-stage feature-selecting method based on mutual information-based best individual feature (MIBIF) and decision tree-based recursive feature elimination (DT-RFE) is utilized for FBACSP features to achieve faster, more accurate, and more robust classification of MI tasks.
3. To verify the feasibility and effectiveness of the proposed FBACSP algorithm, two public benchmark MI-EEG datasets are selected for classification experiments, and the proposed algorithm shows accurate and robust results.
4. With feature visualization, we analyze the differences and connections between the proposed ASP features and the traditional features, thus verifying their complementarity.

The rest of the study is organized as follows: Section 2 describes the methodology of the study, including the extracted FBACSP features and the overall algorithm framework. Section 3 presents the results of the experiment and analyzes the results. Section 4 discusses the proposed method. Finally, Section 5 concludes the study and points out future work.

To facilitate a clear and concise presentation of our research findings, an abbreviations list is provided in Appendix Table A. This list contains the definitions and explanations of any abbreviations or acronyms used in the main text.

## 2 Method

### 2.1 Eature extraction

We propose the FBACSP method as the feature extraction method, and for each frequency band, the features are extracted using CSP and ASP methods separately and merged. As a method that is proven effective on MI tasks, the FBCSP method can extract the energy difference between different leads for different types of MI tasks. On the other hand, the ASP method is used to extract the difference in the total energy of the leads for different types of MI tasks. We use the ASP algorithm as a feature complement to the FBCSP algorithm to improve the overall effect of the algorithm.

#### 2.1.1 Common spatial pattern and filter bank common spatial pattern

CSP algorithm is a spatial filtering feature extraction algorithm for two-class classification tasks, which can extract the spatial distribution components of each class from multi-channel EEG signals. CSP algorithm designs a spatial filter to maximize the difference of variance values between two types of EEG signal matrices after spatial filtering to obtain features with high discrimination. Detailed formulas of CSP are described in Appendix B. For multi-category MI tasks, the one-*vs*-rest (OVR) strategy was used to extend the CSP algorithm (Ang et al., 2008). FBCSP is an extension of the CSP method, which executes CSP algorithms in different sub-bands to obtain FBCSP features. Therefore, for a k-class MI classification task with a number of channels, FBCSP will obtain the features of sub-bands*k*channels in the pre-set sub-bands.

#### 2.1.2 Local best particle swarm optimization

Particle swarm optimization (PSO) is an evolutionary computation technique. Compared with other optimization algorithms, PSO has no restrictions on the form and nature of the objective function and does not require gradient information (Marini and Walczak, 2015). It comes from the study of bird predation behavior. The basic idea of PSO is to find the optimal solution through cooperation and information sharing among individuals in the swarm. PSO simulates a bird in a flock by designing a massless particle with only two attributes: speed and position. The speed represents the moving vector, and the position represents the solution. Each particle searches the optimal solution in the search space independently, which is recorded as the current individual best value. The individual best value is shared with other particles in the whole particle swarm, and the individual best value found is the current global optimal solution of the whole particle swarm. All particles in the PSO adjust their velocity and position according to the current individual extremum found by themselves and the current global optimal solution shared by the entire PSO.

PSO is initialized with a population of random particles as random solutions. The optimal solution is then found by iteration. In each iteration, the particle updates itself by keeping track of two extreme values: (*pbest* and *gbest*). After finding these two optimal values, the particle updates its velocity and position by the following formula:


(1) $$\begin{array}{l} {v_{i}}^{t+1}=\omega^{t}*{v_{i}}^{t}+c_{1}\times r\mathrm{and}\left(t\right)\times \left(\mathrm{pbes}{t_{i}}^{t}-{x_{i}}^{t}\right)\\ \;+c_{2}\times \mathrm{rand}\left(t\right)\times \left(\mathrm{gbes}{t_{i}}^{t}-{x_{i}}^{t}\right) \end{array}$$

(2) $${x_{i}}^{t+1}={x_{i}}^{t}+\max\left({v_{i}}^{t+1},v_{\text{max}}\right)$$

(3) $$\omega^{t}=\frac{\left(\omega_{ini}-\omega_{\mathrm{end}}\right)\left(G-t\right)}{G}+\omega_{\mathrm{end}}$$

Eq. 1 represents the velocity vector update formula, while Eq. 2 represents the position update formula at time *t*. Eq. 3 is the formula for calculating the inertia factor at time *t* using a linearly decreasing weight strategy (LDW).

In Eq. 1, ${v_{i}}^{t}$ is the original velocity vector of particle i at time *t*. $\mathrm{rand}\left(t\right)$ is a random number between 0 and 1 used to increase the randomness of the algorithm. $\omega^{t}$ is the inertia factor, which represents the degree of dependence of the updated velocity vector on the original velocity vector. ${x_{i}}^{t}$ represents the current position of particle *i* while $\mathrm{pbes}{t_{i}}^{t}$ and $\mathrm{gbes}{t_{i}}^{t}$ represent the personal best and global best positions of particle i at time *t*, respectively. $c_{1}$ and $c_{2}$ are the learning factors that represent the degree of learning of individual and global best values. In Eq. 2, the maximum displacement of particles is limited by $v_{\text{max}}$ during each iteration and ${x_{i}}^{t+1}$ is updated iteratively. In Eq. 3, G represents the maximum number of iterations, and $\omega_{ini}$ and $\omega_{\mathrm{end}}$ represent the initial and final values of the inertia weight, respectively.

In this study, the local best PSO algorithm was employed, which differs from the traditional PSO algorithm, in that it defines the global best value as the best value of the k -nearest particles around each particle rather than the true global best value. This results in a longer convergence time but reduces the risk of the algorithm being trapped in local optima. Specifically, the definition of $\mathrm{gbes}{t_{i}}^{t}$ is as follows:


(4) $$\mathrm{gbes}{t_{i}}^{t}=\mathrm{best}\left(i,\mathrm{neighbor}=k\right)$$

Local best PSO is summarized in Algorithm 1:**Algorithm 1: Local Best PSO**
**Input: Particle number NOutput: Global best position gBestSteps:**(1)Initialize particles with random positions *x_i_* and velocities *v_i_* for each *i* in range (N).
(2)Evaluate each particle and set the personal best position *pbest_i_* to *x_i_*.(3)Determine the best neighbor particle k and get *gbest_i_* for each particle *i* by Eq. (4), using a topology like ring or star.
(4)Update *ω* using Eq. (3).
(5)Update particle’s velocity *v_i_* and position *x_i_* using Eq. (1) and Eq. (2) respectively.
(6)While stopping criterion is not met, repeating step(2)-step(5)
(7)**Return** *gbest*


#### 2.1.3 Adaptive spatial pattern

The CSP algorithm enhances the differences in variance values between two types of EEG signal matrices by designing a spatial filter and using it to extract features. These features are in line with the requirements of the ERD/ERS phenomenon for decoding MI tasks, but they also pose some problems. For a binary classification problem of MI EEG signals, consider a data matrix of (*samples, channels,* and *timepoints*). The CSP algorithm can only obtain a fixed (*channels* and *channels*) spatial filter, at most resulting in a (*channels, 1*) feature vector. Moreover, the objective of the spatial filter obtained by the CSP algorithm is only to distinguish the variance values of the EEG signal matrix. Therefore, the features extracted by the CSP algorithm are very limited and not enough to decode MI-EEG signals well. To address this problem, we propose a new spatial-filter-solving paradigm based on the PSO algorithm to complement the CSP spatial filter and named it adaptive spatial pattern (ASP). We combined ASP with CSP features to leverage the strengths of each method and enhance MI-EEG classification. While CSP primarily captures signal discriminability by finding optimal spatial filters that maximize variance differences between classes, it tends to overlook the overall energy distribution within different frequency bands. In this context, ASP was introduced to address this limitation. ASP’s objective is to minimize the intra-class energy matrix and maximize the inter-class energy matrix after spatial filtering, providing insights into the total energy distribution of EEG signals in various frequency bands. This enriches our feature space by offering a holistic view of EEG signal characteristics. Before obtaining a spatial filter, we first establish a standard paradigm of EEG classification based on spatial filtering:**Algorithm 2: EEG classification based on spatial filter**
**Input: Raw training data, raw testing data**
**Output: Predicted testing label**
**Steps:**(1)Preprocess raw training data to obtain processed training data.
(2)Initialize Spatial Filters.
(3)While stopping criterion not met:
a. Apply Spatial Filter to processed training data to obtain filtered training data.
b. Compute the loss between filtered training data and training labels.
c. Minimize the loss by updating the Spatial Filter.
(4)Save the learned Spatial Filters.
(5)Apply the learned Spatial Filters to the processed training data to obtain filtered training data.
(6)Extract features from the filtered training data to obtain train features.
(7)Train a classifier using the train features and training labels.
(8)Save the trained classifier.
(9)Preprocess raw testing data to obtain processed test data.
(10)Apply the learned Spatial Filters to the processed test data to obtain filtered test data.
(11)Extract features from the filtered test data to obtain test features.
(12)Use the trained classifier to predict the labels of the test features.
(13)**Return** Predicted testing label.


Both the CSP algorithm and the proposed ASP in this study conform to the aforementioned standard paradigm for spatial filtering. For the CSP algorithm, the loss function is the difference in variance values between the two types of EEG signal matrices after filtering. For the ASP, we define the loss function as follows:


(5) $$\mathrm{Loss}=\frac{\mathrm{trace}\left(\sum_{k=1}^{K}\sum_{i=1}^{n_{k}}\left(x_{i}-{\overset{-}{x}}_{k}\right){\left(x_{i}-{\overset{-}{x}}_{k}\right)}^{T}\right)}{\mathrm{trace}\left(\sum_{k=1}^{K}n_{k}\left({\overset{-}{x}}_{k}-\overset{-}{x}\right){\left({\overset{-}{x}}_{k}-\overset{-}{x}\right)}^{T}\right)}$$

The numerator represents the within-class matrix of *K*-class MI signals after spatial filtering, while the denominator represents the between-class matrix of *K*-class MI signals after spatial filtering. The objective of the loss function is to make similar signals of the same class of MI after spatial filtering while making different signals of different classes of MI after spatial filtering. x represents the feature extracted after spatial filtering, and in this study, we use energy:


(6) $$x=\log\left(\sum_{t=1}^{\mathrm{timepoints}}|\left(F*X\right)|{}^{2}\right)$$

Where F represents the spatial filter and X represents the EEG signals before the spatial transformation. The process of obtaining the spatial filter in the ASP algorithm is conducted using Algorithm 1. In addition, considering the influence of frequency bands on MI signal energy, we use the same frequency band settings as FBCSP before applying the ASP algorithm, i.e., performing the ASP algorithm in each frequency band. Since the one-*vs*-one (OVO) method can be performed for any two types of MI-EEG signals, more features and matrices can be obtained. In addition, redundant features will be removed by feature selecting; therefore, for the multi-class MI task, we adopt the one-vs-one (OVO) approach to implement Local best PSO is summarized $\mathrm{subbands}*C_{k}^{2}$ features, where subbands is the number of frequency bands and k is the number of task categories.

### 2.2 Feature selection and classifier

For our FBACSP method, we designed a two-stage feature selection strategy. The number of FBCSP features that have not been selected is subbands∗k∗channels , which is much larger than the $\mathrm{subbands}*C_{k}^{2}$ features obtained from FBASP. Moreover, FBCSP features have many redundant features (Lu et al., 2010; Higashi and Tanaka, 2013; Meng et al., 2013; Zhang et al., 2015; Park and Chung, 2019). Therefore, we first use a pre-set mutual information-based best individual feature (MIBIF) method to screen FBCSP features at each frequency band. The purpose of MIBIF is to retain effective FBCSP features while reducing the complexity of subsequent processing. Furthermore, after frequency, band-level MIBIF is used to screen FBCSP features, and the decision tree-based recursive feature elimination (DT-RFE) method is used for the second-stage feature selection of all sub-band FBACSP features. DT-RFE is used to select effective features that are suitable for the subject and to choose spatial filters that are more helpful for classification tasks.

The time complexity of the second stage DT-RFE method is $O\left(n*\mathrm{feature}s^{2}\right)$ , where n is the sample size and features is the number of features. While not using MIBIF, features is the sum of the number of features of FBCSP and FBASP, which is $\mathrm{subbands}*k*\mathrm{channels}+\mathrm{subbands}*C_{k}^{2}$ . MIBIF greatly reduces the time complexity of DT-RFE by reducing the dimension of FBCSP to the same order of magnitude as FBASP and reducing the number of features from $\mathrm{subbands}*k*\mathrm{channels}+\mathrm{subbands}*C_{k}^{2}$ to $\mathrm{constant}*\mathrm{subbands}*C_{k}^{2}$ .

#### 2.2.1 Mutual information-based best individual feature

To reduce the dimensionality of FBCSP features, we used mutual information-based best individual feature (MIBIF) as the feature selection method in each frequency band. MIBIF is a feature selection method based on mutual information. In MIBIF, the n features with the highest mutual information are selected from the feature vectors obtained from the k projection matrices in each frequency band. Mutual information is calculated as follows:


(7) $$I\left(X;Y\right)=\underset{y\in Y}{\sum }\underset{x\in X}{\sum }p\left(x,y\right)\log\left(\frac{p\left(x,y\right)}{p\left(x\right)p\left(y\right)}\right)$$

Here, X and Y are the features and corresponding labels obtained from each OVR projection matrix. After going through MIBIF, FBCSP features can obtain subbands∗k∗n FBCSP features, where subbands is the number of frequency bands, k is the number of task categories, and n is the number of selected features in each projection matrix.

#### 2.2.2 Recursive feature elimination

We used the recursive feature elimination (RFE) method to select spatial filters that would be more helpful for the classification task. FBCSP and FBASP each generated $N_{\mathrm{FBCSP}}$ and $N_{\mathrm{FBASP}}$ spatial filters, and we used RFE to select $N_{\mathrm{FBCSP}-\mathrm{ASP}}$ better spatial filters from them. RFE is a machine learning feature selection algorithm used to build models and reduce computation time, coefficient number, and model complexity. It is an improvement technique for filter methods, especially for feature correlation coefficient screening and filter methods based on L1 regularization. It uses an internal algorithm to recursively eliminate unimportant features. In RFE, at each iteration, a model based on the current best feature subset is constructed. Then, in each iteration, the model sorts each feature according to its importance. Higher-ranked features are retained, and lower-ranked features are recursively removed. The process of collecting important features and iterative model improvement results in the final optimal feature subset. The algorithm implementation of RFE is as follows:**Algorithm 3: Recursive feature elimination**
**Input: FBCSP features, FBASP features, *N***_
**
*FBACSP.*
**
_
**Output: Optimal set of features**
**Steps:**(1)Initialize feature set with all *N_FBCSP_* + *N_FBASP_* features.
(2)Train the model with the current feature set.
(3)Compute the importance of each feature in the trained model.
(4)Drop the feature with the lowest importance score from the feature set.
(5)Repeat step (2)-step (4) for (N_FBCSP_ + N_FBASP_ − N_FBACSP_) times.
(6)**Return** the optimal set of features with the desired number of features.


#### 2.2.3 Decision tree-based recursive feature elimination and random forest

We chose decision tree (DT) as the internal model for RFE, and we used random forest (RF) as the classifier for the model. We selected tree-based models for both feature selection and classification because the features extracted from FBCSP and FBASP are of different orders of magnitude, and tree-based models process features vertically and are not affected by differences in feature magnitude. On the other hand, the loss function used for ASP features is based on the between-class matrix and the within-class matrix, making the tree-based model based on the node value suitable for FBACSP features.

DT is a machine learning classification method based on a tree structure. In DT, classification is performed by iterative splitting of data. Each node from the root node to the leaf node represents a split. For DT, it is necessary to keep the data with the same class as much as possible on one side of the tree. When the data in the leaf node of the tree are all of the same class, classification stops. In this study, the splitting of DT nodes is based on the Gini coefficient:


(8) $$\mathrm{Gini}\left(p\right)=\sum_{k=1}^{k}p_{k}\left(1-p_{x}\right)=1-\sum_{k=1}^{k}p_{x}^{2}$$

Here, k represents the number of classes, $p_{k}$ represents the probability of a particular class in the current category, and $1-p_{x}$ represents the probability that it is not the current class. The larger the Gini coefficient value, the greater the uncertainty of the sample. By calculating the Gini coefficient, we select the attribute that minimizes the Gini coefficient after splitting as the optimal splitting point. Meanwhile, the feature importance in RFE is obtained by calculating the normalized decrease in the Gini coefficient for each feature. For the features used in splitting each node in the decision tree, their feature importance is calculated as follows:


(9) $$\mathrm{Importance}=\frac{N_{t}}{N}*\left(\mathrm{Gini}-\frac{N_{tL}}{N_{t}}*left_{\mathrm{Gini}}-\frac{N_{tR}}{N_{t}}*\mathrm{righ}t_{\mathrm{Gini}}\right)$$

Where N represents the number of samples, $N_{t}$ represents the number of samples in the current node, Gini represents the Gini coefficient of the current node, $N_{tL}$ represents the number of samples in the left child node of the current node, left:Gini represents the Gini coefficient of the left child node of the current node, $N_{tR}$ represents the number of samples in the right child node of the current node, and right:Gini represents the Gini coefficient of the right child node of the current node.

RF is an ensemble learning model based on bagging, with DT as the base classifier. The process of generating decision trees in random forest involves both row and column sampling of the sample data. By randomly selecting a part of the dataset, a tree is generated, and repeating this process generates different decision trees, which together form the random forest. In the output, the final output of the RF is the collective decision results of the decision trees obtained by voting. The training process of the random forest is as follows:**Algorithm 4: Random forest**
**Input: Dataset N, Number of decision trees T, Number of randomly sampled features F**
**Output: Trained model RF**
**Steps:**(1)Initialize an empty list for decision trees, DTs.
(2)Randomly sample F features from N to create a new dataset N′.
(3)Create a new decision tree DT using dataset N′.
(4)Append DT to DTs.
(5)Repeat step (2)-step (4) for T times.
(6)Create the random forest classifier RF by uniformly selecting from the decision trees in DTs.
(7)**Return** trained RF.


### 2.3 FBACSP

**Figure 1:**
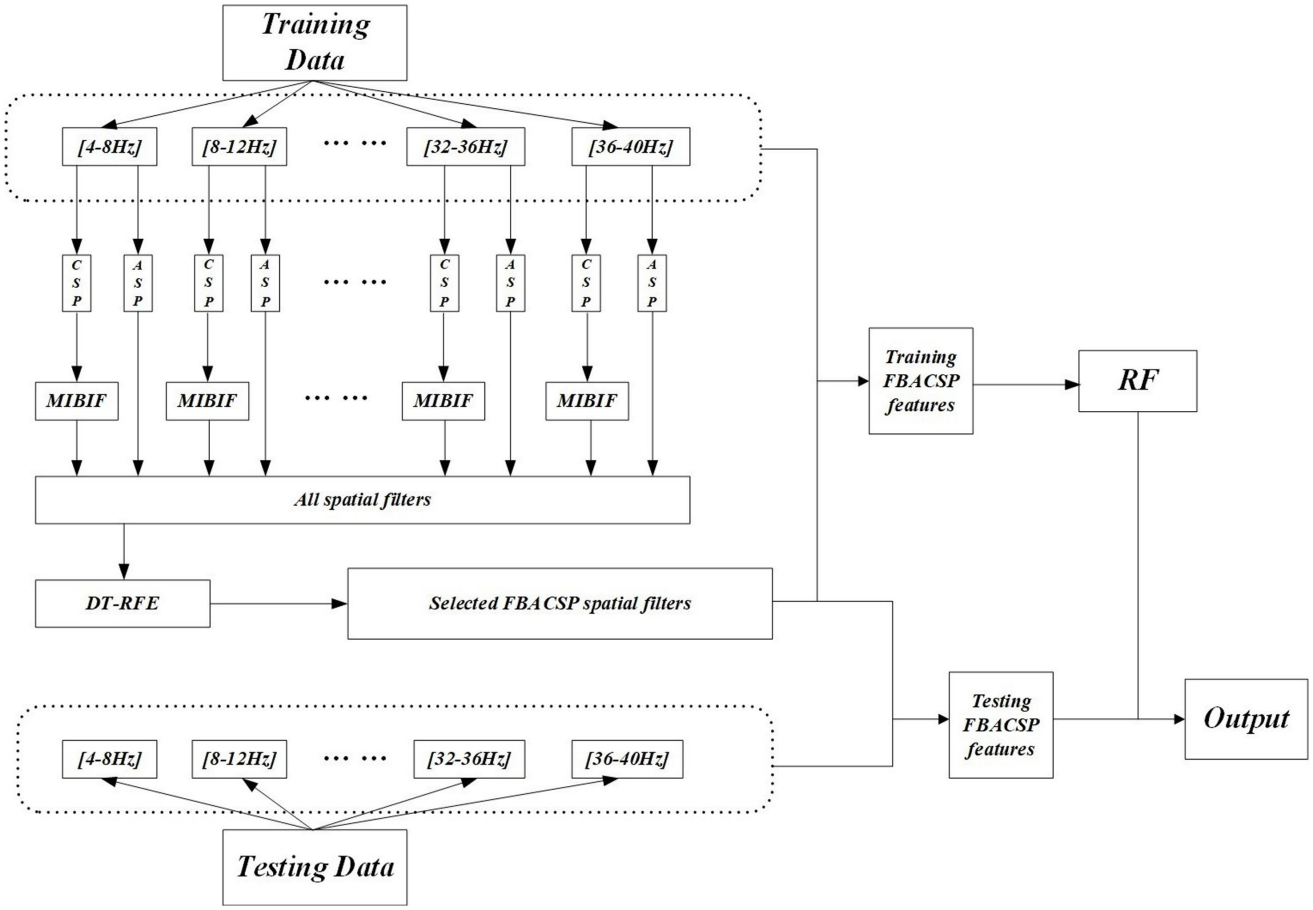
The overall framework for the proposed FBACSP algorithm.

In summary, we integrate the feature extraction, feature selection, and classifier discussed above, continuing previous research (Ang et al., 2008). Same as settings in baseline, nine sub-bands were set, ranging from 4–8 Hz, 8–12 Hz to 36–40 Hz. The training and testing framework of the FBACSP algorithm is shown in the Figure 1.

During the training stage, the original signal used for training is first filtered into nine sub-bands. CSP and ASP features are then separately computed on each sub-band. The CSP features on each sub-band are subjected to the first round of feature selection using the MIBIF method to coarsely reduce the total feature dimension. This can greatly reduce the computation of the subsequent DT-RFE method while retaining effective CSP features. The ASP spatial filters and the selected CSP spatial filters on each sub-band are merged. The merged CSP and ASP features on each sub-band are then pooled together, resulting in a total of $\mathrm{subbands}*\left(C_{k}^{2}+k*n\right)$ feature vectors. These feature vectors are sent to DT-RFE for dimension reduction, and the optimal number of features after dimension reduction is determined through 5-fold validation on the training set. Then, the classifier is trained on these features and saved. During the testing stage, the original signal used for testing is filtered into the same sub-bands, and the saved FBACSP spatial matrices are used to extract the corresponding features in each sub-band. These features are classified by the trained classifier to obtain the final output.

## 3 Materials

### 3.1 Datasets

To verify the feasibility and effectiveness of our proposed FBACSP algorithm, we conducted experiments on two publicly available benchmark datasets from BCI Competition IV: dataset 2a and dataset 2b (Tangermann et al., 2012). Both datasets contain MI-EEG signals collected from nine different subjects. Dataset 2a contains four MI tasks (left hand, right hand, feet, and tongue), while dataset 2b contains two MI tasks (left hand and right hand). Each subject in dataset 2a has two sessions, and each session has 72 trials across four categories. Each subject in dataset 2b had five sessions, with two sessions containing 120 trials and the remaining three sessions containing 160 trials. The EEG signals in both datasets were filtered by a 0.5-Hz to 100-Hz band-pass filter and a 50-Hz notch filter, and the artifacts were marked and removed by experts. Before applying the algorithms, all EEG signals underwent re-referencing. As suggested in Schirrmeister et al. (2017), high-pass filtering can serve as a means to address artifacts, so we did not take any other steps for artifact-removing. Filtering methods employed in this study utilized Chebyshev second-order filters. The experimental paradigm for performing MI tasks in both datasets is shown in Appendix Figure C1. Initially, a warning beep and a cross were presented on the screen to keep the subjects focused. Then, an arrow prompt was displayed on the screen to guide the classification of the MI task. After a 1-s prompt, the subjects began to perform the MI task according to the guidance, which lasted for 4 s. Upon completing the MI task, the subjects entered a rest period. It should be noted that the last three sessions of dataset 2b included smiley feedback, but this feature was not specifically addressed in this study.

### 3.2 Experimental setups

For both datasets, we chose to utilize EEG signals recorded from 0.5 to 3.5 s after the cue as the input for the algorithm. This selection allows us to capture essential neural activity associated with the task while minimizing noise and irrelevant data. We omitted the data from the 0.5 s before and after this window to exclude potential artifacts or unrelated neural activity (Fang et al., 2022). In dataset 2a, for any given subject, we used the first session as the training set and the second session as the testing set. In dataset 2b, for any given subject, we used the first three sessions as the training set and the last two sessions as the testing set. The two datasets are described in Table 1. The distribution of EEG electrodes in both two datasets is described in Appendix Figure C2.

**Table 1:** Description of datasets.

	Number of electrodes	Sampling rate	Selected motor imagery duration for each sample	Number of training samples/each subject	Number of testing samples/each subject
Dataset 2a	22	250 Hz	3,000 ms	288	288
Dataset 2b	3	250 Hz	3,000 ms	400	320

To compare the performance of the method, we conducted experiments on each subject in two datasets to obtain the classification accuracy and calculated the average accuracy and standard deviation on each dataset. The hyperparameter settings required for the experiment involve the PSO algorithm, the MI-based dimensionality reduction after FBCSP, the RFE algorithm, and the RF classifier. Among them, some hyperparameters are obtained by the mesh parameter tuning method with 10-fold validation on the training set. The hyperparameter settings are described in Appendix Table C1. Among them, the PSO parameter settings are obtained according to the conclusion of research (Juneja and Nagar, 2016) to achieve the global optimum faster and avoid the local optimum as much as possible.

## 4 Results

### 4.1 Comparison results of motor imagery classification

To compare the accuracy of our proposed algorithm, we used the FBCSP+SVM algorithm and the FBASP+RF as two baseline methods. Additionally, we compared our proposed algorithm FBCASP with Deep ConvNet (Schirrmeister et al., 2017), Shallow ConvNet (Schirrmeister et al., 2017), EEGNet (Lawhern et al., 2018), C2CM (Sakhavi et al., 2018), and STNN (Sun et al., 2022). The results on dataset 2a and 2b are presented in the Tables 2, 3.

**Table 2:** Results on dataset 2a.

	FBCSP	FBASP	Deep ConvNet	Shallow ConvNet	EEGNet	C2CM	STNN	FBACSP
A1	77.4	82.3	86.6	79.5	85.0	**87.5**	82.3	**87.5**
A2	54.2	47.6	62.3	56.3	56.6	**65.3**	47.6	59.0
A3	69.8	84.3	89.9	88.9	81.7	90.3	88.9	**90.6**
A4	56.3	66.6	65.6	**80.9**	66.4	66.7	60.8	67.4
A5	46.9	55.5	55.2	57.3	54.9	62.5	**66.7**	63.2
A6	52.1	49.6	48.5	53.8	59.6	45.5	**57.9**	57.3
A7	83.0	62.5	86.1	91.7	**92.3**	89.6	85.8	83.3
A8	60.4	75.7	78.4	81.2	75.7	**83.3**	77.1	80.2
A9	68.4	72.1	76.1	79.2	74.8	79.5	80.9	**83.0**
Mean	63.17	66.24	72.10	74.31	71.89	74.47	72.20	**74.61**
SD	12.19	12.74	14.83	14.54	13.28	15.33	13.48	12.13

Each numerical entry represents the percentage accuracy of a specific method on a given subject within Dataset 2a. The labels A1 through A9 correspond to the nine subjects within Dataset 2a. “Mean” denotes the average percentage accuracy across all nine subjects, while “SD” signifies the standard deviation of the mean accuracy calculated over the nine subjects. The bold values in a single row represent the highest classification accuracy among all methods in a subject.

**Table 3:** Results on dataset 2b.

	FBCSP	FBASP	Deep ConvNet	Shallow ConvNet	EEGNet	C2CM	STNN	FBACSP
B1	70.3	69.4	72.0	74.2	73.8	**74.8**	85.0	74.4
B2	55.4	58.9	57.0	55.8	56.7	61.3	75.2	**62.5**
B3	55.6	60.9	64.9	55.4	64.5	65.5	**68.2**	61.2
B4	94.7	88.5	94.4	91.6	93.2	94.4	**98.9**	97.1
B5	80.6	85.8	89.9	88.7	81.9	86.7	75.0	**90.3**
B6	80.0	83.0	83.3	83.3	85.8	**87.5**	82.0	85.0
B7	74.1	76.5	78.1	74.1	72.7	79.4	83.2	**83.8**
B8	79.7	86.1	90.8	88.6	**91.5**	89.6	79.5	88.6
B9	76.3	79.2	77.9	72.8	72.5	81.7	79.0	**87.8**
Mean	74.08	76.47	78.70	76.09	76.96	80.10	80.7	**81.19**
SD	12.47	10.41	12.48	11.95	12.20	11.13	8.50	11.79

Each numerical entry represents the percentage accuracy of a specific method on a given subject within Dataset 2b. The labels B1 through B9 correspond to the nine subjects within Dataset 2b. “Mean” denotes the average percentage accuracy across all nine subjects, while “SD” signifies the standard deviation of the mean accuracy calculated over the nine subjects. The bold values in a single row represent the highest classification accuracy among all methods in a subject.

From the results in Tables 2, 3, it can be seen that the proposed FBACSP method has achieved satisfactory performance on datasets 2a and 2b, reaching accuracies of 74.6 and 81.2%. The accuracy of the FBACSP algorithm is significantly improved than FBCSP using a statistical student *t*-test (*p* < 0.05) on both datasets, resulting in an accuracy improvement of 11.44 and 7.11% on two datasets, respectively. Interestingly, when comparing the performance of the two baselines on the two datasets, we found that some subjects were particularly suited to CSP or ASP features. For example, in dataset 2a, subject a3 achieved significantly better results with FBASP (84.3%) than with FBCSP (69.8%), while subject a7 showed the opposite trend, with FBCSP (83.0%) outperforming FBASP (62.5%). Compared to the two baselines, the proposed FBACSP algorithm achieved better classification performance by not only adding additional spatial filters but also by removing redundant features using the DT-RFE algorithm. Moreover, on average, we found that the proposed FBACSP algorithm outperforms traditional deep learning models such as EEGNet. Additionally, it shows results that are comparable to those of recent models such as EEGNet, C2CM, and STNN. In addition, since recent deep learning models have introduced more complex network architectures, these models are more complex in interpretability. For further validation, we performed 10-fold cross-validation to assess the model’s intra-session performance. In this experiment, we utilized the first session of data from both datasets for experimentation. The experiment was conducted using a 10-fold cross-validation with class balance. The detailed results for dataset 2a and dataset 2b can be found in Appendix Tables A2, A3.

### 4.2 Results analysis

We analyze the results from three perspectives. First, we conducted an analysis of the FBACSP features before performing DT-RFE feature selection at the individual level. We explored the performance of the ASP and CSP features in different frequency bands using a three-dimensional histogram of mutual information. Next, we visualized the features to investigate the effectiveness of the FBACSP algorithm. Finally, we investigated the contribution of the FBACSP features to classification at the model level. Subject A3 and subject B5 were selected as representative for datasets 2a and 2b for detailed analysis.

#### 4.2.1 Feature-level analysis by mutual information

To investigate the performance of ASP and CSP features in different frequency bands, as well as to demonstrate the effectiveness of the ASP algorithm, we analyzed the FBACSP features of each subject before DT-RFE feature selection using a mutual information-based approach. Mutual information was calculated using Eq. 7. For each subject in dataset 2a and dataset 2b, we calculated the mutual information of each feature and plotted them in the form of a three-dimensional bar graph.

**Figure 2:**
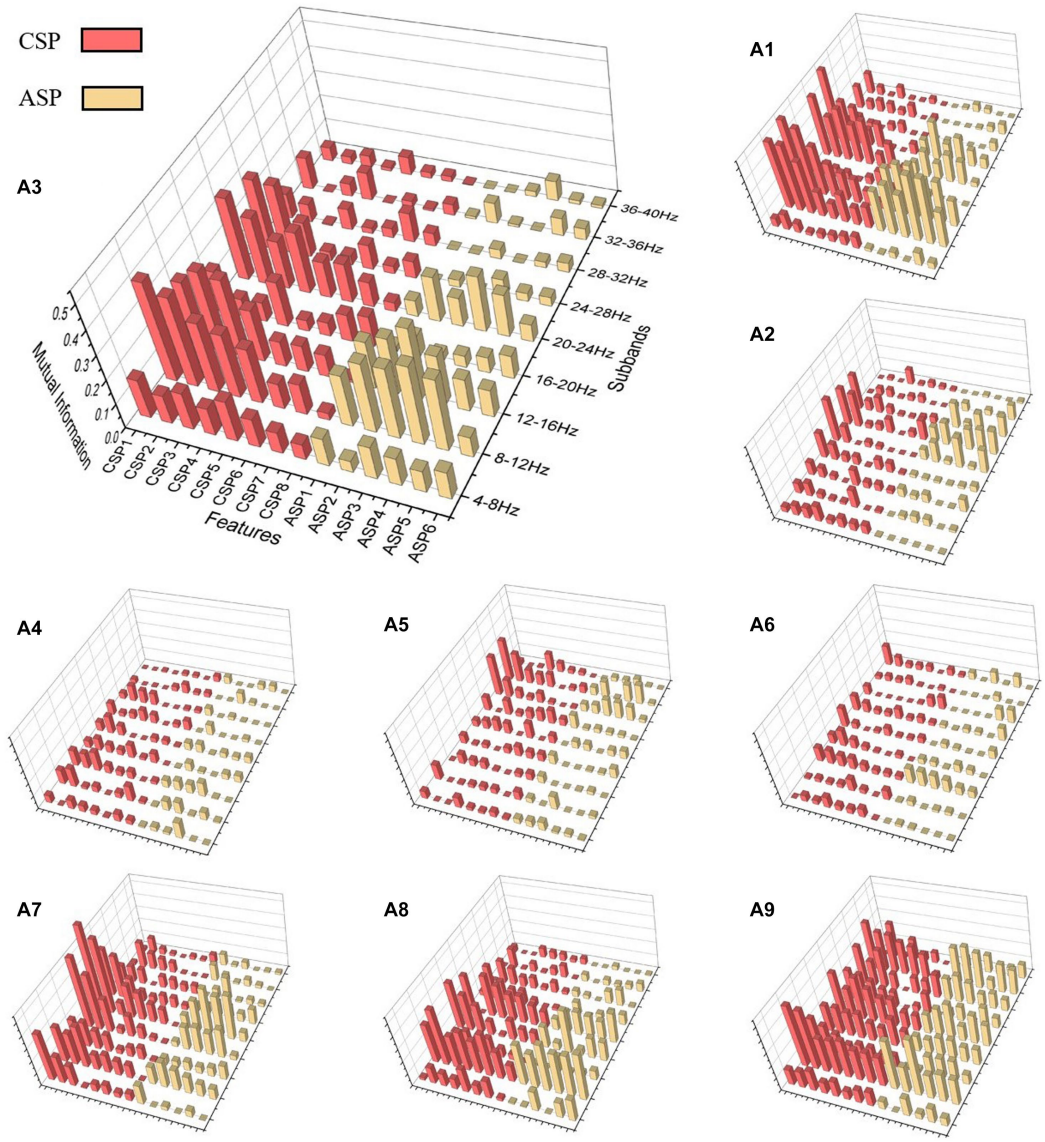
Mutual information 3D plot for dataset 2a. CSP features are in red and ASP features are in yellow. The position of the cylinder on the plane represents the type and frequency band of the feature, and the height of the cylinder represents the mutual information value of the feature. The subject number represented by each subfigure is marked at the top left of the subfigure.

**Figure 3:**
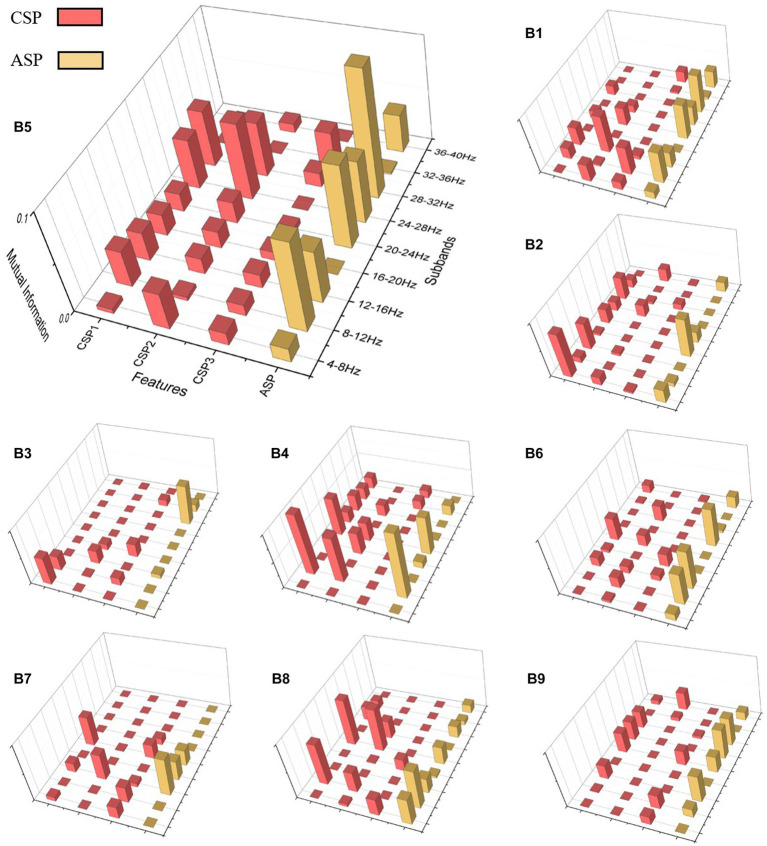
Mutual information 3D plot for dataset 2b.

Figures 2, 3 present the mutual information of all FBCSP and FBASP features for each subject in datasets 2a and 2b. The CSP and ASP features are marked in red and yellow. We use A3 and B5 as examples for the four-class and binary-class analysis. For A3, the mutual information values in the frequency bands of 8–12 Hz, 12–16 Hz, and 20–24 Hz are higher than others. For subject A3, the mutual information values of CSP features are slightly higher than those of ASP features in all frequency bands. This indicates that CSP features perform better on subject A3. However, for subject B5, ASP features have higher mutual information values than CSP features in the 8–16 Hz and 20–23 Hz frequency bands. Considering that CSP features extract differential features through spatial transformation, ASP features extract overall energy features through spatial transformation; this suggests that the suitable types of features for subjects during MI tasks are different.

In summary, the sub-bands where different subjects’ features perform well are not the same, but FBCSP and ASP features generally have similar changes in mutual information at the frequency band level. On the other hand, although FBCSP has higher mutual information values than ASP in most sub-bands, ASP still has equally high mutual information values. Even in subjects in dataset 2b, the ASP features in the same sub-band may have higher mutual information values.

#### 4.2.2 Feature visualization

**Figure 4:**
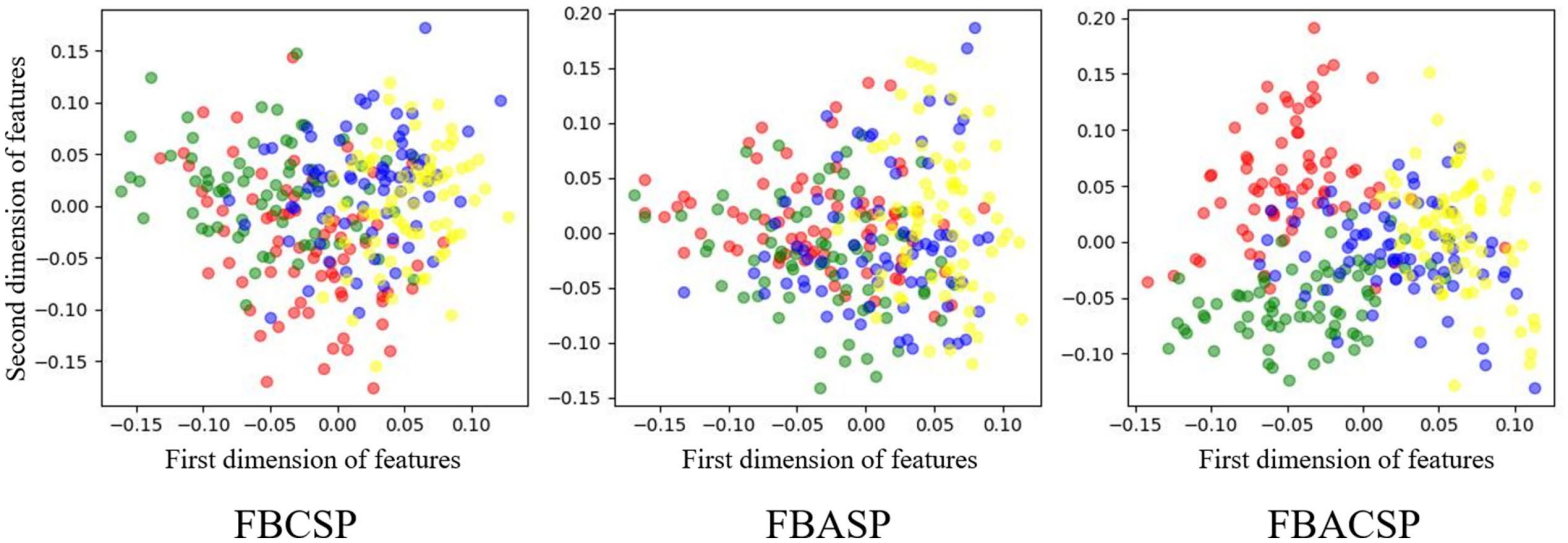
Feature visualization on dataset 2a. The colors representing the left hand, right hand, foot, and tongue are in red, green, blue, and yellow.

**Figure 5:**
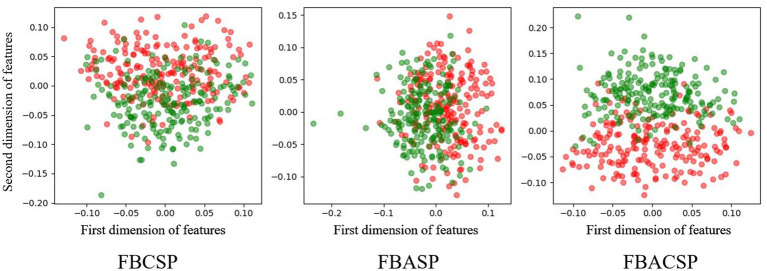
Feature visualization on dataset 2b. The colors representing the left hand and right hand are red and green.

To explore the optimization effects of the FBACSP algorithm on features, we utilized the dimensionality reduction visualization tool t-SNE (Van der Maaten and Hinton, 2008) for feature visualization. Figures 4, 5 show the visualization of different classes of features under the optimal FBACSP features for both datasets. For horizontal comparison, we also present the results of the classic FBCSP features and the use of FBASP features only. In terms of visualization, the t-SNE tool was used to reduce the features of each group of EEG signals to two dimensions and displayed in scatter plots.

The results from Figures 4, 5 demonstrate that the proposed FBACSP features outperform traditional FBCSP features for datasets 2a and 2b. The selected FBACSP features exhibit better separability in t-SNE visualization for both the four classes in dataset 2a and for two classes in dataset 2b. It means that the discriminability of the FBCSP features, which serve as the baseline, is weaker than the selected FBACSP features. On the other hand, neither using only FBCSP nor only FBASP features can fully distinguish between classes. However, after combining FBACSP features, the features are better clustered by class, which demonstrates the complementarity of CSP and ASP features.

#### 4.2.3 Comparison of optimization algorithms

**Figure 6:**
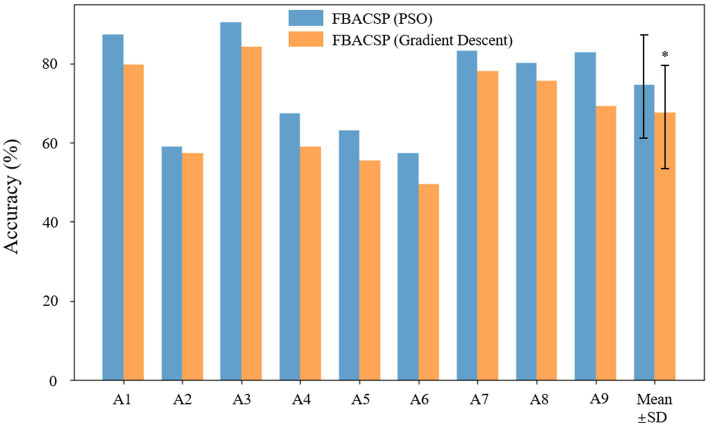
Comparison of optimization algorithms. Where “*” indicates statistical significance (*p* < 0.05).

To compare the efficacy of the local best PSO with an alternative optimization approach, we implemented the Gradient Descent algorithm for spatial filter optimization. Gradient Descent is a widely used optimization technique in machine learning and signal processing. We performed a series of experiments with Gradient Descent to evaluate its impact on spatial filter performance, thus providing insights into the feasibility of using this traditional approach. In this section, we present a comprehensive comparison of the results obtained using PSO and Gradient Descent for spatial filter optimization. The evaluation metrics using classification accuracy are reported and analyzed. This comparison will shed light on the relative advantages and disadvantages of the two optimization techniques, offering valuable insights into the optimal choice for spatial filter design in MI-EEG classification. Figure 6 shows the comparison of classification accuracy results using two different optimization algorithms on dataset 2a.

In the comparative analysis between PSO and Gradient Descent for spatial filter optimization, our results revealed notable differences in performance. We observed that the utilization of Gradient Descent led to significantly lower classification accuracy and kappa value in comparison to the results achieved using PSO. Several factors contribute to this observed discrepancy. Gradient Descent, being a gradient-based optimization technique, relies on local gradient information and may suffer from issues such as getting stuck in local optima. In contrast, PSO, a population-based optimization method, often explores a wider search space, allowing it to escape local optima more effectively. This property enables PSO to discover spatial filters that are better suited for MI-EEG decoding, resulting in superior classification performance. The findings emphasize the importance of selecting an appropriate optimization algorithm for spatial filter design within the ASP framework. While Gradient Descent has been widely employed in various optimization tasks, our results indicate that, in the context of MI classification, PSO stands out as a more effective choice.

#### 4.2.4 Feature contribution analysis by Shapley values

To substantiate the enhancement achieved by incorporating ASP into FBCSP, we employ Shapley additional (SHAP) values as a powerful analytical tool. SHAP values enable us to comprehensively investigate the influence and contribution of each individual feature toward the final classifier’s performance. SHAP is a model explanation method from game theory (Lundberg and Lee, 2017), which is proven to achieve interpretability for machine learning (Lamens and Bajorath, 2023). Considering a situation where a coalition of players co-create value and reap benefits, SHAP gives a calculation method to distribute the benefits. SHAP allocates expenditure to players according to their contribution to the total expenditure. For a regression model, all input variables contribute to the final prediction, so every variable is a player in the coalition. The prediction is the co-created value of the coalition. The importance of variables, namely SHAP values, is measured by how much they contribute to the prediction. For sample x , the SHAP value of variable j is calculated by the following formula:


(10) $$\Phi_{j}\left(\mathrm{val}\right)=\underset{S\subseteq \left\{x_{1},\ldots ,x_{p}\right\}\left\{x_{j}\right\}}{\sum }\frac{|S|!\left(p-S-1\right)!}{p!}\left(\mathrm{val}\left(S\cup \left\{x_{j}\right\}\right)-\mathrm{val}\left(S\right)\right)$$

Where val is a specific model and $\Phi_{j}\left(\mathrm{val}\right)$ is the SHAP value of variable j with this model. S is a subset of input variables. |S| is the number of variables in subset S . p is the total amount of variables in the prediction model. The global SHAP value of variable j is the sum of absolute SHAP values of j among all samples. Therefore, for an *n* classification task, we will get SHAP values for each of the *n* classes.

**Figure 7:**
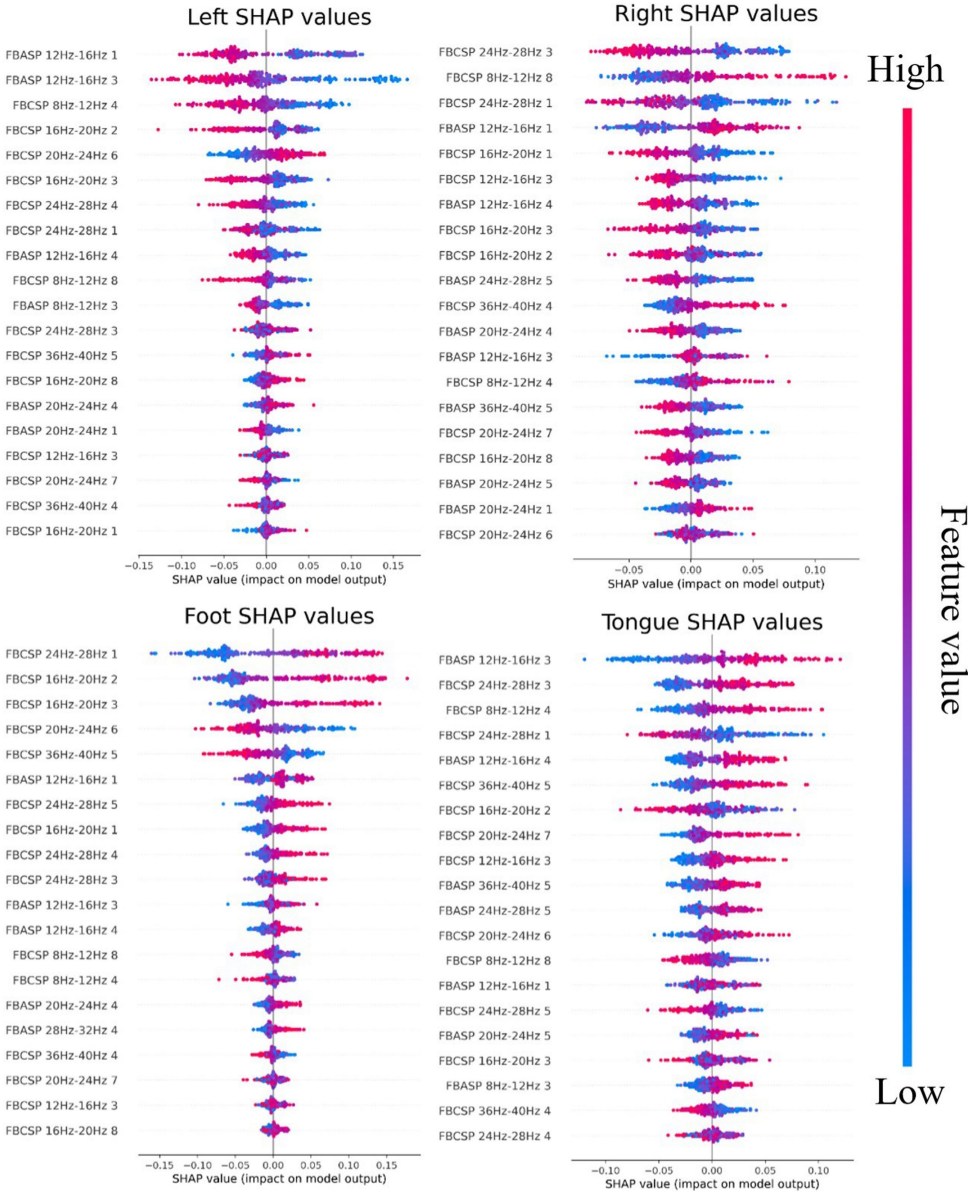
SHAP values for FBACSP features on dataset 2a. Each subplot represents the contribution of features to the classification of a type of MI task. For each subplot, the ranking of features is from top to bottom according to the mean absolute value of SHAP in all samples.

Figure 7 illustrates the SHAP values of the FBACSP method for MI four-class classification. In any row of the subplot, each point represents a sample, and its lateral position is the SHAP value calculated for the corresponding feature on that sample. The color of the point represents the ranking of the corresponding feature value in all samples, ranging from blue–purple–red. It is evident that when distinguishing between right hand and foot movements, CSP-based features exhibit a significant advantage. However, when it comes to distinguishing between left-hand and tongue motor imagery, the inclusion of ASP features plays a more substantial role in enhancing the classifier’s performance. A notable example can be observed when examining the SHAP values in Figure 7 for distinguishing left-hand motor imagery. Specifically, the feature labeled ‘FBASP 12 Hz-16 Hz 1’ exhibits a distinct transition point at 0, indicating its minimal redundancy in relation to the samples. Moreover, as the value of the ‘FBASP 12 Hz-16 Hz 1’ feature increases, the tendency is toward classification as non-left-hand motor imagery, while a lower value tends to result in the classification of left-hand motor imagery. Compared with the best FBCSP-based feature in the same plot, “FBCSP 8 Hz-12 Hz 4,” which also has good separability, cannot provide valuable information to the classifier for samples with feature values in the center. Considering the calculation process of CSP and ASP algorithms, it can be inferred that for the left-hand discrimination task, the spatial filters obtained by the ASP algorithm have better performance than those obtained by the CSP algorithm.

**Figure 8:**
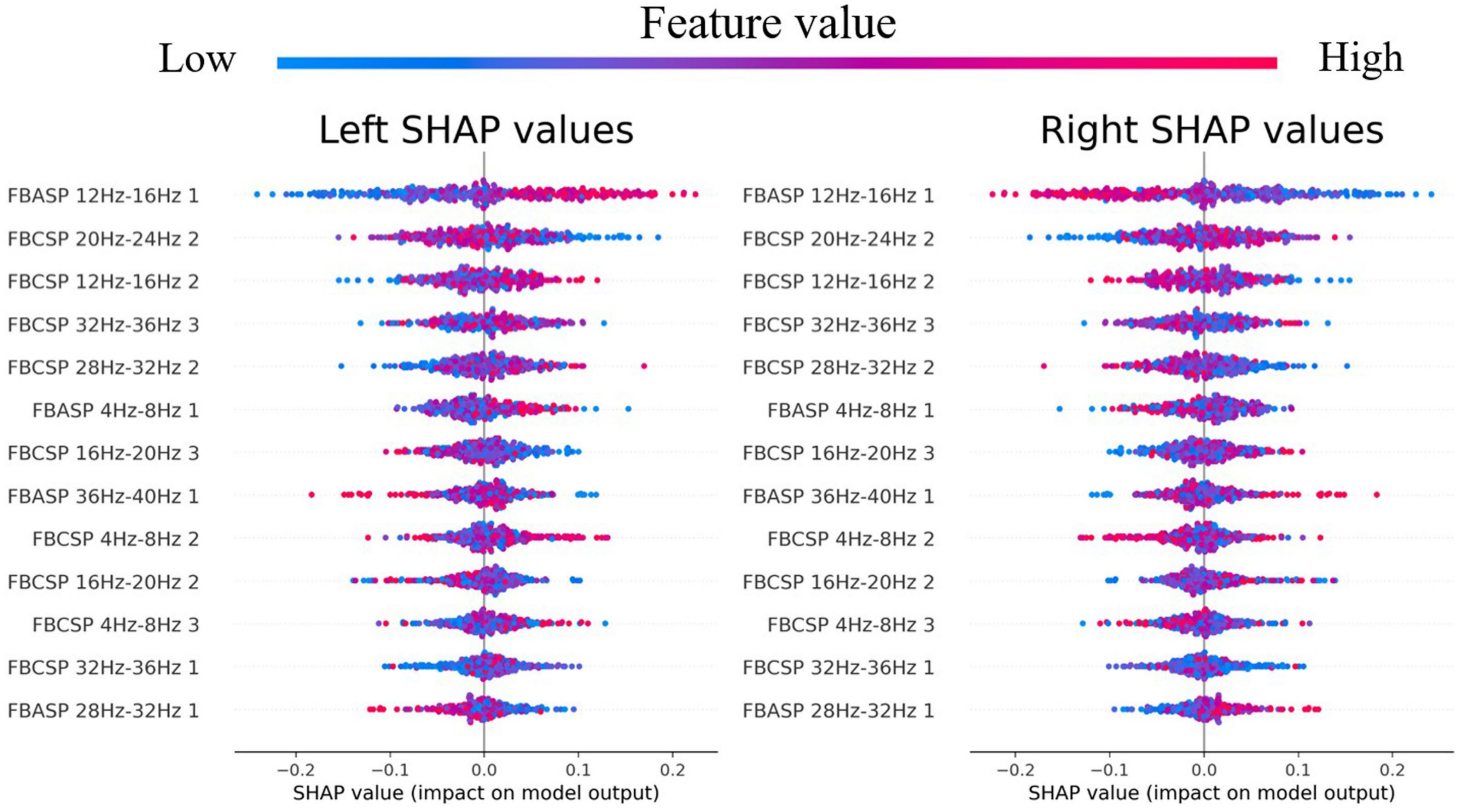
SHAP values for FBACSP features on dataset 2b.

Figure 8 illustrates the SHAP values of the FBACSP method for binary classification in dataset 2b. For a binary classification task, the discriminative contribution of any feature to both classes is symmetric. As shown in Figure 8, the ASP feature in the 12–16 Hz frequency band has the greatest effect on the classifier. On the other hand, apart from the ASP feature in the 12–16 Hz frequency band, most of the selected features are CSP features. From Figure 8, it can be inferred that after the transformation by the corresponding ASP spatial filters, the energy of the 12–16 Hz frequency band of the EEG signal is larger during left-hand motor imagery than that during right-hand motor imagery. The SHAP values of binary classification indicate that although FBASP can produce better features, FBCSP features still have a significant effect.

## 5 Discussion

This study proposes a novel spatial filter-based EEG signal feature extraction method, called the ASP method, and designs an FBACSP method for classifying MI EEG signals. The proposed algorithm outperforms traditional machine learning-based algorithms in the classification of MI and achieves excellent results on two datasets.

From the results of Figures 5, 6, we find that the features selected by the FBACSP algorithm show better classification performance on datasets 2a and 2b. Although the training process of FBACSP is more complex, the spatial filters selected by the FBACSP method can directly act on the filtered EEG signals during the testing phase. If the trained classification model is used for online applications, for each sample, spatial filtering can be performed on different frequency bands and energy features can be extracted for direct use in MI classification, resulting in higher classification performance.

To further investigate the relationship between ASP features and CSP features and demonstrate that the features selected by FBACSP are the most discriminative, we employed 3D mutual information plot, t-SNE, and SHAP values to analyze and visualize the features. The 3D mutual information plot was used to visualize the relationship between ASP and CSP features at the feature level. T-SNE was used to analyze the differences in FBCSP/FBASP/FBACSP features from an intuitive perspective and transform them into 2D space. SHAP values were used to analyze the contribution of FBACSP features to the model. In the experimental results, we displayed the results of three different methods: FBCSP, FBASP, and FBACSP. The 3D mutual information plot calculates the mutual information between CSP and ASP features on each frequency band and the labels, which can intuitively display the relationship between ASP and CSP features. In t-SNE analysis, we intuitively found that FBACSP features improve upon both FBCSP and FBASP features, which validates the effectiveness of the FBACSP method and the complementarity between ASP and CSP features. SHAP values demonstrate the contribution of ASP and CSP features in different frequency bands to each class classification by calculating the contribution value of each feature to Eq. 10, which further validates the effectiveness of ASP features in the classifier.

The proposed FBACSP algorithm has three advantages:

1. Extendibility: Our Algorithm 1 breaks the tradition that only the CSP algorithm can be used as a spatial filter and proposes a customizable process for calculating brain signal spatial filtering features. The ASP feature proposed in this study is an instance of Algorithm 2, which uses Algorithm 1 as a spatial filtering calculation method and Eqs. 5, 6 as loss functions. For further research, algorithms for finding spatial filters and loss functions for spatial filtering can be modified. Therefore, the ASP feature is very flexible and has strong expandability for different EEG signal classification tasks.
2. Generalization: The FBACSP algorithm has a certain generalization ability for MI-EEG signal classification. Compared with the baseline algorithm, our proposed algorithm has greatly improved average classification accuracy for all subjects on two datasets. In addition, although the training process of the FBACSP algorithm is complex, once the CSP and ASP spatial filters on each frequency band are determined, they can be applied to the EEG signals of the entire subject collected on a certain acquisition device, showing good practicality in MI classification. Unlike deep learning models that require a large number of training experiments, the proposed FBACSP algorithm can be trained and applied in a small number of EEG experiments. Therefore, it has a broad application prospect and potential in wearable EEG devices, wireless transmission EEG devices, and many other application scenarios.
3. Interpretability: The proposed FBACSP algorithm uses RF as a classifier, which enables us to analyze more detailed mutual information processes. Meanwhile, interpretable methods, such as SHAP values applicable to machine learning, can also be used for analysis. Such analysis can reveal which type of features have better performance on each subject and the contribution of different features to classification for each subject, as shown in Figures 6, 7. This interpretability is of great value to the research in the field of MI-BCI.

## 6 Conclusion

This study introduces a novel spatial filter paradigm, adaptive spatial pattern (ASP), which differentiates itself from traditional CSP methods by emphasizing the optimization of energy distribution within and between different motor imagery tasks. The FBACSP method combines ASP spatial filtering with CSP features across all frequency bands, and we employ the local best PSO algorithm to enhance spatial filter optimization, extending beyond CSP capabilities. Our approach streamlines feature sets by utilizing mutual information for preliminary CSP feature screening and merging them with ASP features through the DT-RFE method.

Our findings reveal that FBACSP features outperform FBCSP features and achieve competitive results with state-of-the-art methods when evaluated on two publicly available EEG datasets. The classification accuracy of the proposed method has reached 74.61 and 81.19% on datasets 2a and 2b. Detailed analyses employing mutual information, t-SNE, and SHAP provide valuable insights into feature responses. Our future endeavors will focus on the practical application of this algorithm in online motor imagery-based brain–computer interfaces for stroke therapy. We acknowledge that while our algorithm enhances baseline classification accuracy, opportunities for further refinement exist, including the exploration of ASP method variants, improved spatial filter optimization techniques, and the investigation of more effective loss functions.

## Data availability statement

Publicly available datasets were analyzed in this study. This data can be found here: https://www.bbci.de/competition/iv/results/index.html#dataset2a, https://www.bbci.de/competition/iv/results/index.html#dataset2b.

## Author contributions

XX: Conceptualization, Formal analysis, Methodology, Software, Visualization, Writing – original draft. YW: Data curation, Investigation, Writing – review & editing. TS: Data curation, Investigation, Writing – review & editing. JH: Project administration, Supervision, Writing – review & editing. GK: Project administration, Supervision, Writing – review & editing.
