# Supplementary material for: Improved motor imagery classification using adaptive spatial filters based on particle swarm optimization algorithm
Source: Front Neurosci. 2023 Dec 13;17:1303648. doi: 10.3389/fnins.2023.1303648 (PMC10773845; doi:10.3389/fnins.2023.1303648)
Supplement: Supplementary file 1 [file Table_1.docx]

## Appendix A

**Table A.1** Abbreviations list.

| Abbreviation | Definition |
| --- | --- |
| ASP | Adaptive spatial pattern |
| BCI | Brain-computer interface |
| CSSP | Common spatial-spectral pattern |
| CSP | Common spatial pattern |
| DFBCSP | Discriminative filter bank common spatial pattern |
| DT | Decision tree |
| DT-RFE | Decision tree-based recursive feature elimination |
| EEG | Electroencephalogram |
| ERD/ERS | Event-related desynchronization and event-related synchronization |
| FBCSP | Filter bank common spatial pattern |
| FBACSP | Filter bank adaptive and common spatial pattern |
| FBRCSP | Filter bank regularized common spatial pattern |
| LDW | Linearly decreasing weight strategy |
| LCR-SW-CSP | Common spatial pattern based on the longest continuous repeated sliding window |
| LRFCSP | Local region frequency common spatial pattern |
| MI | Motor imagery |
| MIBIF | Mutual information-based best individual feature |
| PTFBCSP | Penalized Time-frequency band common spatial pattern |
| PSO | Particle swarm optimization |
| RF | Random forest |
| RCSP | Regularized common spatial pattern |
| SCSSP | Separable common spatial-spectral pattern |
| SHAP | Shapley additional |
| SNR | Signal-to-noise ratio |
| SVM | Support vector machine |
| SWCSP | Spectrally weighted common spatial pattern |
| t-SNE | t-Distributed Stochastic Neighbor Embedding |
| TFCSP | Time-frequency common spatial pattern |
| TSGSP | Temporal constrained sparse group spatial pattern |

**Table A.2** Intra-session results on dataset 2a. Each numerical entry represents the 10-fold cross validation percentage accuracy of a specific method on a given subject within Dataset 2a. The labels A1 through A9 correspond to the nine subjects within Dataset 2a. "Mean" denotes the average percentage accuracy across all nine subjects, while "SD" signifies the standard deviation of the mean accuracy calculated over the nine subjects.

|  | FBCSP | FBASP | Deep ConvNet | Shallow ConvNet | EEGNet | C2CM | STNN | FBACSP |
| --- | --- | --- | --- | --- | --- | --- | --- | --- |
| A1 | 65.6 | 83.4 | 77.1 | 84.4 | 84.2 | **93.1** | 85.5 | 90.1 |
| A2 | 56.9 | 57.2 | 56.0 | 62.2 | 61.2 | 65.9 | 53.1 | **66.4** |
| A3 | 65.3 | 91.1 | 91.0 | 90.3 | **96.2** | 93.1 | 87.8 | 94.3 |
| A4 | 62.2 | 76.9 | **74.3** | 74.0 | 66.6 | 69.8 | 61.9 | 68.2 |
| A5 | 26.0 | 50.6 | **78.8** | 70.5 | 60.2 | 66.6 | 68.2 | 70.5 |
| A6 | 47.9 | 59.2 | **68.4** | 56.9 | 56.7 | 48.2 | 60.4 | 60.8 |
| A7 | 75.4 | 68.1 | 91.0 | 91.7 | 85.8 | 91.5 | 86.9 | **92.0** |
| A8 | 64.2 | 70.7 | 81.3 | 84.1 | **84.8** | **84.8** | 77.2 | **84.8** |
| A9 | 62.2 | 78.3 | 82.3 | 82.9 | 78.9 | 81.9 | 83.0 | **83.1** |
| Mean | 58.32 | 70.61 | 77.77 | 76.48 | 74.95 | 77.21 | 73.80 | **78.91** |
| SD | 13.65 | 12.47 | 10.95 | 12.38 | 11.78 | 11.26 | 12.41 | 11.68 |

**Table A.3** Intra-session results on dataset 2b. Each numerical entry represents the 10-fold cross validation percentage accuracy of a specific method on a given subject within Dataset 2b. The labels B1 through B9 correspond to the nine subjects within Dataset 2b. "Mean" denotes the average percentage accuracy across all nine subjects, while "SD" signifies the standard deviation of the mean accuracy calculated over the nine subjects.

|  | FBCSP | FBASP | Deep ConvNet | Shallow ConvNet | EEGNet | C2CM | STNN | FBACSP |
| --- | --- | --- | --- | --- | --- | --- | --- | --- |
| B1 | 60.1 | 72.8 | 78.8 | 76.3 | 80.0 | 83.6 | **93.0** | 84.3 |
| B2 | 56.4 | 62.0 | 72.1 | 66.1 | 73.2 | 70.9 | **85.6** | 70.3 |
| B3 | 55.9 | 67.1 | **84.1** | 78.8 | 83.1 | 73.0 | 76.0 | 71.0 |
| B4 | 95.0 | 95.6 | 97.5 | 97.2 | 97.8 | **98.6** | 97.6 | **98.6** |
| B5 | 78.4 | 89.3 | **98.6** | 98.8 | 97.5 | 96.0 | 82.2 | 97.0 |
| B6 | 78.8 | 91.2 | 85.3 | 86.9 | 90.9 | 93.8 | 92.3 | **95.0** |
| B7 | 78.1 | 84.0 | 93.4 | 88.8 | **94.1** | 88.5 | 92.3 | 92.5 |
| B8 | 88.4 | 83.5 | 95.0 | 93.1 | 93.1 | 96.8 | 87.4 | **97.1** |
| B9 | 75.9 | 80.1 | 88.8 | 85.3 | 89.1 | 89.9 | 87.1 | **94.9** |
| Mean | 74.22 | 81.30 | 88.20 | 85.67 | 88.76 | 87.90 | 88.19 | **88.97** |
| SD | 12.99 | 11.28 | 8.97 | 10.55 | 8.37 | 9.59 | 6.24 | 10.56 |

## Appendix B

For a two-class EEG classification task, the two categories are denoted as matrices X1 and X2 respectively, and their shapes are *channels***time-samples*. The mathematical model of composite source is used to describe the EEG signal. Under the condition of ignoring the influence of noise, the two matrices can be described as follows:

$$\begin{aligned} X_{1}=\left[ C_{1} C_{M} \right]\left[ \begin{matrix} S_{1} \\ S_{M} \end{matrix} \right]\#\left( 1 \right) \end{aligned}$$

$$\begin{aligned} X_{2}=\left[ C_{2} C_{M} \right]\left[ \begin{matrix} S_{2} \\ S_{M} \end{matrix} \right]\#\left( 2 \right) \end{aligned}$$

Here, $S_{1}$and $S_{2}$ represent the source signals that are linearly independent of each other in the two types of EEG signals, and $S_{M}$ is the source signal common to the two tasks. $C_{1}$ and $C_{2}$ are composed of the common spatial patterns corresponding to the respective sources of $S_{1}$ and $S_{2}$, respectively. Each spatial pattern is a vector of $channels*1$, and the physical meaning of this vector represents the distribution weight of the signal caused by a single source signal on all channels. $C_{M}$ then represents the source signal common to the two types corresponding to $S_{M}$. The purpose of CSP is to find an optimal set of spatial filters for projection that maximizes the difference of variance values between two types of signals. The sum R of the normalized average covariance matrices of $X_{1}$ and $X_{2}$ is denoted as follows:

$$\begin{aligned} R=\bar{R_{1}}+\bar{R_{2}}=\frac{X_{1}{X_{1}}^{T}}{trace\left( X_{1}{X_{1}}^{T} \right)}+\frac{X_{2}{X_{2}}^{T}}{trace\left( X_{2}{X_{2}}^{T} \right)}\#\left( 3 \right) \end{aligned}$$

Where ${X_{1}}^{T}$ and ${X_{2}}^{T}$are the transpose of $X_{1}$ and $X_{2}$, respectively, $trace$ denotes the trace of the matrix, with $\bar{R_{1}}$ and $\bar{R_{2}}$ denote the average covariance matrices of the covariance matrices $R_{1}$ and $R_{2}$ in the respective class experiments, respectively. The mixture spatial covariance matrix is obtained by eigenvalue decomposition:

$$\begin{aligned} R=U\lambda U^{T}\#\left( 4 \right) \end{aligned}$$

Where $U$ is the matrix of eigenvectors and $\lambda$ is the diagonal matrix formed by the corresponding eigenvalues. The eigenvalues are sorted in descending order, and the whitening value matrix $P$ is:

$$\begin{aligned} P=\sqrt{\lambda^{-1}}U^{T}\#\left( 5 \right) \end{aligned}$$

The covariance matrices $R_{1}$ and $R_{2}$ are transformed as follows:

$$\begin{aligned} S_{1}=PR_{1}P^{T}\#\left( 6 \right) \end{aligned}$$

$$\begin{aligned} S_{2}=PR_{2}P^{T}\#\left( 7 \right) \end{aligned}$$

After that, $S_{1}$ and $S_{2}$ are decomposed by principal components:

$$\begin{aligned} S_{1}=B_{1}\lambda_{1}{B_{1}}^{T}\#\left( 8 \right) \end{aligned}$$

$$\begin{aligned} S_{2}=B_{2}\lambda_{2}{B_{2}}^{T}\#\left( 9 \right) \end{aligned}$$

Here, $B_{1}$ and $B_{2}$ are the eigenvector matrices of $S_{1}$ and $S_{2}$, $\lambda_{1}$ and $\lambda_{2}$ are the corresponding eigenvectors of $B_{1}$ and $B_{2}$, respectively, and the sum of $\lambda_{1}$ and $\lambda_{2}$ is the identity matrix. That is, the sum of eigenvalues of the two types of matrices is always$1$. Therefore, the covariance matrix of each trial data is whitened and projected along its whitened total covariance matrix to obtain the optimal projection transformation matrix, that is, the spatial filter $W$ is:

$$\begin{aligned} W=B^{T}P\#\left( 10 \right) \end{aligned}$$

For two types of data $X_{1}$ and $X_{2}$, the corresponding CSP feature vector is represented by:

$$\begin{aligned} \left\{ \begin{aligned} Z_{1}=W*X_{1} \\ f_{1}=\frac{var\left( Z_{1} \right)}{sum\left( var\left( Z_{1} \right) \right)} \end{aligned} \right.\#\left( 11 \right) \end{aligned}$$

$$\begin{aligned} \left\{ \begin{aligned} Z_{2}=W*X_{2} \\ f_{2}=\frac{var\left( Z_{2} \right)}{sum\left( var\left( Z_{2} \right) \right)} \end{aligned} \right.\#\left( 12 \right) \end{aligned}$$

Therefore, after CSP transformation, the spatial domain feature vector of $channels*1$ can be obtained.

## Appendix C

**Fig. C.1.** Experimental paradigm.


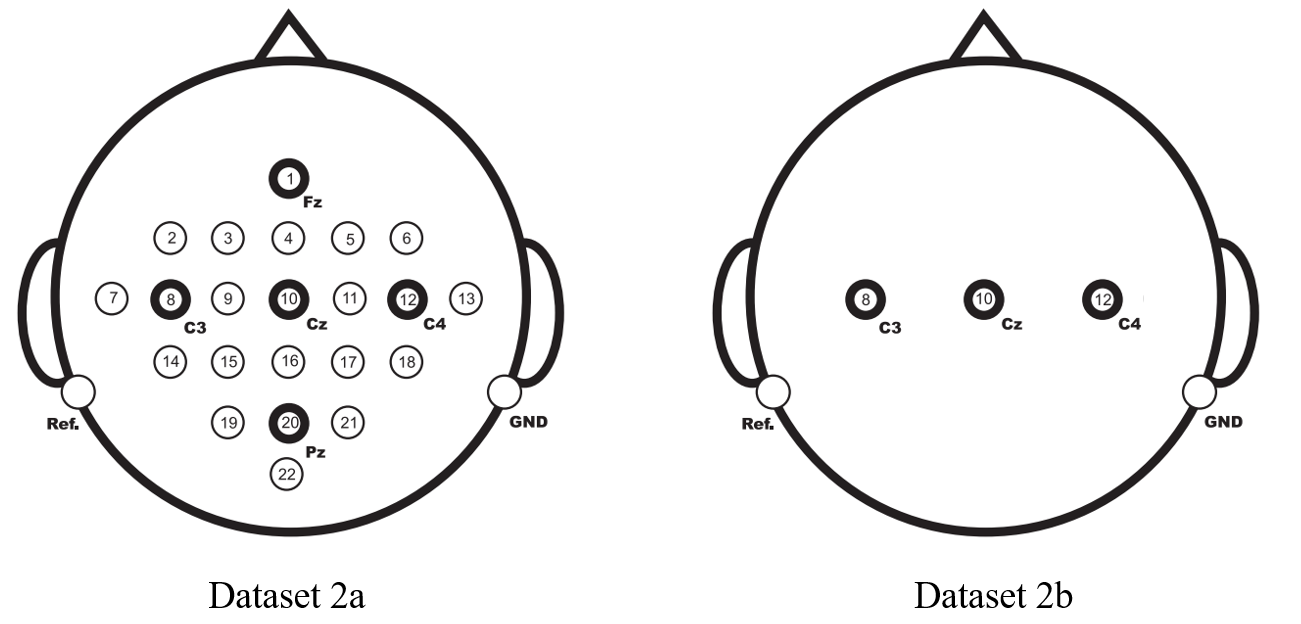


**Fig. C.2.** Distribution of electrodes.[43]

**Table C.1** Description of hyperparameters.

|  | Hyperparameters | Range or value for Dataset 2a | Range or value for Dataset 2b |
| --- | --- | --- | --- |
| Local PSO | *c_1_*  *c_2_*  *w*  *k*  Particle lower bound  Particle upper bound  Number of particles  Iterations | 2  2  0.729  20  -100  100  100  2000 | 2  2  0.729  15  -100  100  60  2000 |
| MIBIF | Number of selected features for each subband | 8 | None |
| DT-RFE | Max depth  Number of selected features | None  5 to 125 | None  1 to 36 |
| RF | Number of DTs  Max depth  Min samples split  Min samples leaf | 5 to 200  1 to 20, None  1 to 10  1 to 5 | 5 to 200  1 to 20, None  1 to 10  1 to 5 |
